# Calcifications discales intervertébrales chez l’enfant: à propos de deux cas

**DOI:** 10.11604/pamj.2016.25.34.10543

**Published:** 2016-09-27

**Authors:** Eitel Igor Kouamo, Merouane Nour, Jean Marie Gennar, Jean Marc Guillaume, Elie Choufani, Thierry Merrot, Jean Pierre Alessandrni, Kathia Chaumoitre, Michel Panuel, Namane Doumbouya, Patrice Guemaleu

**Affiliations:** 1 Service de Chirurgie Infantile, Hôpital Nord Chemin des Bourrelys, 13015 Marseille, France; 2 Service de Radiologie, Hôpital Nord Chemin des Bourrelys, 13015 Marseille, France; 3 Service de chirurgie pédiatrique, Centre Hospitalier de Lens, 99 route de la Bassée 62300 Lens, France

**Keywords:** Herniated intervertebral calcifications, child, torticollis, Intervertebral disc calcifications, child, torticollis

## Abstract

Les calcifications discales sont le plus souvent révélées chez l'enfant, par des crises douloureuses rachidiennes. Leur localisation cervicale, semble être la plus fréquente. Cependant, les autres localisations sont moins symptomatiques et pourraient ainsi passer inaperçues. Nous rapportons deux observations d'enfants hospitalisés pour des calcifications discales cervicales symptomatiques. Il s'agit : du cas d'une fillette âgée de quatre ans et demi ayant présenté un torticolis, révélateur de la migration d'une calcification discale jusque-là asymptomatique ; et de celui du garçon âgé de 05 ans venu pour cervicalgie aigue et chez qui une calcification discale a été retrouvée. Leur traitement était essentiellement conservateur. Les calcifications discales intervertébrales de l'enfant sont une pathologie rare dont l'étiopathogénie reste inconnue. Leur diagnostic ne doit pas se faire sans un examen neurologique attentif et une imagerie devant des douleurs rachidiennes inexpliquées chez l'enfant.

## Abstract (en)

Disc calcifications are usually revealed in children by pain crises that affect the spine. The most common location for disc calcifications is the cervical spine. However, other locations are less symptomatic and they may well go unnoticed. We here report two cases of children hospitalized with symptomatic cervical disc calcifications. The first case involves a girl aged four and a half suffering from torticollis revealing the migration of a previously asymptomatic disc calcification ; the second case, instead, involves a 5-year old boy presenting for acute neck pain and in whom calcification disc was detected. Treatment was essentially conservative. Intervertebral disc calcifications in children are a rare condition whose etiopathogenesis remains unknown. In the case of children with unexplained back pain the diagnosis should not be made without careful neurological examination and imaging tests.

## Introduction

Du symptôme de torticolis aigu chez l'enfant, peuvent être diagnostiquées des affections graves. Un examen clinique minutieux et l'appoint d'un bilan iconographique bien conduit, permettent de s'orienter vers une étiologie moins grave et rare : les calcifications discales inter vertébrales (CDI). Celles-ci sont liées un changement dégénératif du disque intervertébral, et intéressent le plus souvent, le rachis cervical [1]. Cette pathologie même si elle n'est pas toujours retenue au premier plan du fait de sa rareté, il est utile de savoir déjouer le piège diagnostique afin d'instaurer un traitement adéquat. A travers deux observations d'enfant ayant présenté une calcification discale nous discuterons les perspectives dans les démarches diagnostiques. Le but est d'établir, les particularités diagnostiques, thérapeutiques et pronostiques de cette entité pathologique chez l'enfant.

## Patient et observation

**Figure 1:**
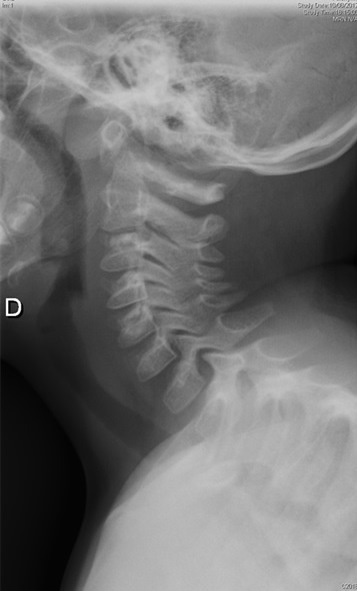
Radiographie du rachis cervical incidence de profil ou l’on voit au niveau des vertèbres C2C3 des calcifications discales étagées des disques intervertébraux chez le patient 1

**Figure 2:**
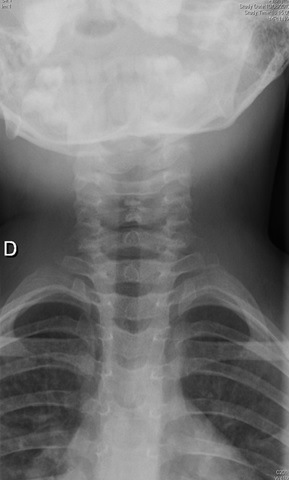
Radiographie du rachis cervical incidence de Face ou l’on voit au niveau des vertèbres C2C3 des calcifications discales étagées des disques intervertébraux

**Figure 3:**
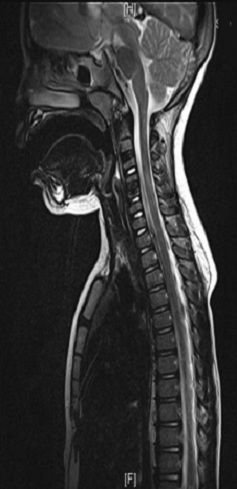
Mise en évidence des calcifications chez le patient 1 à l’IRM sans signe de compression médullaire associé

**Observation N°1:** Il s'agissait d'une fillette de quatre ans demis, qui s'est présentée aux urgences pour un torticolis non fébrile. Elle était connue d'un ATCD de dermatite atopique. L'anamnèse retrouvait des cervicalgies modérées, associées à un torticolis gauche, évoluant depuis deux jours dans un contexte non traumatique. L'examen somatique reproduisait, une douleur à la palpation des épineuses et des muscles para vertébraux du rachis cervical, associée à une nette limitation de la mobilité du cou. Le reste de l'examen clinique (neurologique et oto-rhino-laryngologique) était sans particularité. Le bilan biologique révélait, une hyperleucocytose à 16 900/mm^3^, une CRP à 31mg/l, une fibrine à 5,11 g/l. La calcémie, la phosphorémie ainsi que les phosphatases alcalines étaient de valeurs normales. Les radiographies standards du rachis cervical F/P, ont mis en évidence des calcifications étagées au niveau disques intervertébraux à hauteur de C-C3 (Figure 1). En complément, une TDM et une IRM rachidienne ont précisés leur localisation au centre du disque, au sein de C2/C3 et C4/C5 sans compression médullaire (Figure 2, Figure 3). L'évolution a été rapidement favorable sous traitement conservateur associant, repos, antalgiques et minerve souple.

**Figure 4:**
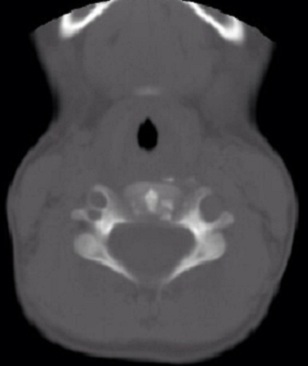
Calcifications discales chez le cas 2 à hauteur de C3 sur une coupe TDM

**Figure 5:**
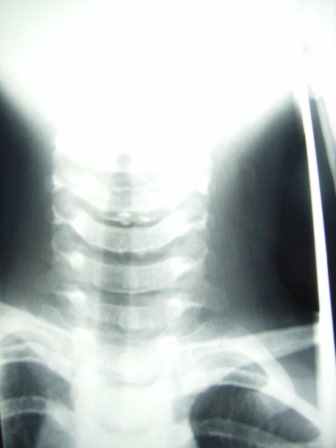
Calcifications inter discales à hauteur de C3 sur l’incidence de radiographie de face chez le patient 2

**Figure 6:**
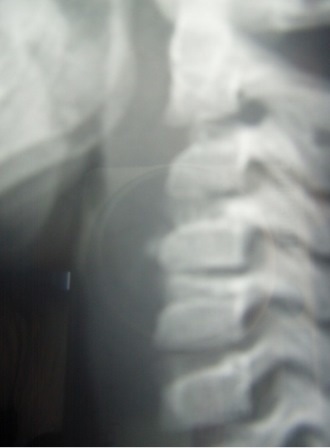
Calcifications inter discales à hauteur de C3 sur l’incidence de radiographie de profil chez le patient 2

**Observation N°2:** Il s'agit d'un garçon de cinq ans, vu aux urgences pour une cervicalgie de survenue brutale sans notion de traumatisme ni d'hyperthermie. Il avait été suivi pour une hypotonie congénitale. La cervicalgie a persistée malgré l'intervention de l'ostéopathe. Vu aux urgences, ou une radiographie standard du rachis cervical F/P (Figure 4) et une immobilisation cervicale par un collier mousse avec une prescription d'antalgiques (paracétamol une dose poids toutes les 08 heures en systématique pendant 05 jours). Le collier a été gardé pendant 10 jours. Revu en consultation à J10, la persistance de la symptomatologie a conduit à réaliser un bilan complémentaire. C'est ainsi qu'une calcification discale (intra et extra) a été retrouvé à hauteur de C4 et C5 à la TDM (Figure 5, Figure 6). Les leucocytes étaient à 15200/mm^3^, la CRP à 20 mg/l les phosphatases alcalines : 189 UI/l, calcium : 97 mg/l, le phosphore 50 mg/l, la vitamine D 35.80 mmol/l Revu 3 mois après, la cervicalgie avait spontanément régressée et à la radiographie standard de contrôle, il n y avait plus d'image de calcification.

## Discussion

Les calcifications discales sont constituées d'une pâte molle contenant des cristaux d'hydroxyapatite de calcium. Elles touchent le nucleus pulposus, et plus rarement la totalité du disque vertébral [2]. Depuis sa première description en 1924 par Baron [3], chez l'enfant, à cette date on compte plus de 300 cas décrits dans la littérature [4]. Environ 100 cas de formes symptomatiques [5] et environ 15% de formes asymptomatiques [2]. Leur fréquence globale est encore mal connue. Les cas rapportés restent exclusivement ceux des enfants à peau dite blanche [6]. L'étiologie des calcifications discales n'est pas connue de nos jours. Aucun facteur prédisposant n'est attribué à cette pathologie. Plusieurs facteurs ont été incriminés comme le traumatisme [7], l'infection [8], la cause auto-immune [9] l'hypervitaminose D et les chondrocalcinoses sont parfois incriminées. Même si ces facteurs n'ont pas été retrouvés chez nos 2 patients, leur étiologie reste en général inconnue [4, 7] mais chez les enfants, les hyperparathyroïdies et l'hémochromatose sont tout aussi souvent citées dans les étiologies. L'âge moyen de leur survenue est de sept à huit ans avec des extrêmes de sept jours de vie à 20 ans. Il existe une légère prédominance masculine [6]. Les C.D.I symptomatiques peuvent survenir à n'importe quel niveau du rachis, avec une très nette prédominance à l'étage cervical. L'espace le plus touché est C6-C7. Les C.D.I asymptomatiques se situent essentiellement à l'étage thoracique [10]. Les symptômes les plus souvent retrouvés, regroupent des douleurs cervicales, des dorsalgies, une diminution de la mobilité rachidienne, une contracture, une raideur para-vertébrale pouvant être responsable d'un torticolis, une fièvre modérée, qui représentent 75% des cas [6] Alors que les formes asymptomatiques 25% des cas, sont des découvertes d'examen systématique en particulier à l'occasion de radiographie pulmonaire [6]. Dans l'anamnèse, on met en évidence dans les jours précédents une notion de traumatisme dans 30% des cas, ou une infection respiratoire haute dans environ 15 % des cas, mais il semble que cette association reste purement fortuite.

Les examens biologiques montrent fréquemment une hyperleucocytose modérée, une élévation de la vitesse de sédimentation, une hyper-protéinorachie. Les radiographies mettent en évidence des calcifications rondes ou ovales intéressent le nucleus pulposus. Lorsque la scintigraphie est réalisée, on retrouve une hyperfixation au niveau de l'espace calcifié ce qui correspond aux premiers symptômes douloureux ; celle-ci reste hyper fixant même après la disparition de la calcification [11, 12]. Ainsi, il est important dans la démarche diagnostique devant un torticolis, d'éliminer certains diagnostics différentiels dont le plus important de par sa gravité est la spondylodiscite. Une infection du disque intervertébral, peut se présenter avec des symptômes habituels des C.D.I, une limitation de la mobilité et une raideur rachidienne. Mais en cas de spondylodiscite les douleurs sont beaucoup plus importantes, intéressant le plus souvent la région lombaire. L'examen radiographique montre une atteinte d'un seul étage intervertébral (Figure 4) où l'on peut voir un rétrécissement de cet espace, une érosion des plateaux vertébraux adjacents sans aucune calcification visible [13]. L'évolution des C.D.I est marquée par la disparition de la crise douloureuse dans un délai d'un mois dans 70% des cas [14]. Cette crise est, accompagnée d'une fragmentation de la calcification et annonce sa disparition radiologique (Figure 6) dans un délai de 28 jours à six mois. Lorsque survient une protrusion du disque, la disparition de la calcification eut s'observer dans les même délais. On peut ainsi décrire des facteurs de persistance de la calcification : pas de crise vraie, localisation plutôt dorsale, calcification homogène non fragmentée, et des facteurs de disparition des calcifications : crise véritable, atteinte cervicale, calcification fragmentée ou protruse. Au niveau radiographique, on remarque très fréquemment des troubles de développement des corps vertébraux accompagnant les C.D.I, dans le cadre d'une crise douloureuse typique. Ainsi, on remarque une évolution radiographique des lésions dans le temps avec initialement un pincement ou un élargissement de l'espace intervertébral, des érosions des plateaux, des géodes à leur niveau et un retard d'apparition du listel marginal. Succèdent ensuite une phase de reconstruction, du sixième au douzième mois après la crise et la disparition de la calcification et on observe soit un aspect normal, soit un trouble de croissance en hauteur du ou des corps vertébraux (Figure 1). Lors du suivi au long cours d'enfants ayant présenté des C.D.I, il a été noté des dorsalgies chroniques, des scolioses. Tous ces éléments plaident en faveur d'une affection rachidienne intéressant le corps vertébral, le cartilage et le disque, malgré l'évolution spontanément bénigne et transitoire de la calcification [7, 15].

Le pronostic des C.D.I de l'enfant est excellent. Les symptômes régressent de quelques jours à quelques semaines après le début du traitement symptomatique. De rares observations font état de complications. Le traitement est conservateur. Il associe antalgiques et anti-inflammatoires non stéroïdiens, myorelaxants et réduction de l'activité physique. En cas de protrusion discale, il est admis que les indications de la chirurgie de décompression médullaire doivent être réservées aux cas de radiculalgies sévères, de déficits sensitivomoteurs significatifs et persistants en rapport avec une compression radiculaire ou médullaire. La chirurgie consiste en une laminectomie de décompression, une discectomie et une fusion intervertébrale [14]. Dans la littérature, on ne relève que dix cas d'enfants traités chirurgicalement dont la plupart avant 1980 [16]. Actuellement l'attitude est beaucoup plus conservatrice accompagnée d'une surveillance de l'évolution symptomatique [17]. Malgré un très bon pronostic, un cas clinique a été décrit en 2002 mettant en évidence la possibilité de survenue de séquelles au long cours au sein de cette pathologie réputée comme bénigne. Il s'agit d'un enfant de 11 ans, qui a présenté des dorsalgies accompagnées d'un syndrome de compression médullaire en T2-T3 : une chirurgie avec une laminectomie de décompression a été réalisée. Néanmoins le suivi à trois ans a mis en évidence un déficit sensitivomoteur persistant [18]. Par contre deux cas avec une symptomatologie neurologique, ont été rapportés par Bajard, traités seulement par un traitement conservateur dont l'évolution était bonne sans séquelles [19]. A ce jour, le traitement des C.D.I n'est pas bien codifié. Il n'existe pas de golden standard chez l'enfant [20]. Le traitement conservateur a montré son efficacité dans certains cas chez l'enfant [19].

## Conclusion

Les calcifications discales intervertébrales de l'enfant sont une entité monomorphe rare dont l'évolution bénigne dans la grande majorité des cas justifie un traitement purement conservateur. L'atteinte dorsale est souvent asymptomatique et cervicale symptomatique. Le diagnostic est radiologique et il n'est pas nécessaire de recourir à d'autres examens complémentaires ; Le diagnostic différentiel est avec une spondylodiscite infectieuse. L'étiopathogénie est inconnue, mais il semble qu'elle soit la conséquence d'un trouble de la vascularisation des disques et plateaux vertébraux, possiblement en rapport avec des phénomènes périnataux. Les complications et les récidives sont exceptionnelles ; Par ailleurs, un suivi de ces enfants au long cours est nécessaire.
